# The botanical origin and antioxidant, anti-BACE1 and antiproliferative properties of bee pollen from different regions of South Korea

**DOI:** 10.1186/s12906-020-03023-1

**Published:** 2020-07-25

**Authors:** Yuchi Zou, Jian Hu, Wenting Huang, Liyun Zhu, Mingjie Shao, Confidence Dordoe, Young-Joon Ahn, Dongxue Wang, Yeli Zhao, Ye Xiong, Xue Wang

**Affiliations:** 1 The First Affiliated Hospital of Wenzhou Medical University, Wenzhou, Zhejiang, 325000 China; 2 School of Pharmaceutical Sciences, Wenzhou Medical University, Wenzhou, Zhejiang, 325035 China; 3 School of the First Clinical Medical Sciences, Wenzhou Medical University, Wenzhou, Zhejiang, 325035 China; 4 College of Agriculture and Life Sciences, Seoul National University, Seoul, 151-921 South Korea

**Keywords:** Bee pollen, Botanical origin, Antioxidant, Anti-BACE1, Antiproliferative, Biological properties

## Abstract

**Background:**

Bee pollen (BP) has been used as a traditional medicine and food diet additive due to its nutritional and biological properties. The potential biological properties of bee pollen vary greatly with the botanical and geographical origin of the pollen grains. This study was conducted to characterize the botanical origin and assess the antioxidant effects of ethanol extracts of 18 different bee pollen (EBP) samples from 16 locations in South Korea and their inhibitory activities on human β-amyloid precursor cleavage enzyme (BACE1), acetylcholinesterase (AChE), human intestinal bacteria, and 5 cancer cell lines.

**Methods:**

The botanical origin and classification of each BP sample was evaluated using palynological analysis by observing microscope slides. We measured the biological properties, including antioxidant capacity, inhibitory activities against human BACE1, and AChE, and antiproliferative activities toward five cancer cell lines, of the 18 EBPs. In addition, the growth inhibitory activities on four harmful intestinal bacteria, six lactic acid-producing bacteria, two nonpathogenic bacteria, and an acidulating bacterium were also assessed.

**Results:**

Four samples (BP3, BP4, BP13 and BP15) were found to be monofloral and presented four dominant pollen types: *Quercus palustris*, *Actinidia arguta*, *Robinia pseudoacacia*, and *Amygdalus persica*. One sample (BP12) was found to be bifloral, and the remaining samples were considered to be heterofloral. Sixteen samples showed potent antioxidant activities with EC_50_ from 292.0 to 673.9 μg mL^− 1^. Fourteen samples presented potent inhibitory activity against human BACE1 with EC_50_ from 236.0 to 881.1 μg mL^− 1^. All samples showed antiproliferative activity toward the cancer cell lines PC-3, MCF-7, A549, NCI-H727 and AGS with IC_50_ from 2.7 to 14.4 mg mL^− 1^, 0.9 to 12.7 mg mL^− 1^, 5.0 to > 25 mg mL^− 1^, 2.7 to 17.7 mg mL^− 1^, and 2.4 to 8.7 mg mL^− 1^, respectively. In addition, total phenol and flavonoid contents had no direct correlation with antioxidant, anti-human BACE1, or antiproliferative activities.

**Conclusion:**

Fundamentally, Korean bee pollen-derived preparations could be considered a nutritional addition to food to prevent various diseases related to free radicals, neurodegenerative problems, and cancers. The botanical and geographical origins of pollen grains could help to establish quality control standards for bee pollen consumption and industrial production.

## Background

There are many products, such as propolis, royal jelly, beeswax and bee pollen (BP), that are harvested from honey bees (*Apis mellifera* L.) [1]. Bee pollen is collected by worker honey bees from the pollen grains of flower anthers in plants and mixed with a secretion from their salivary glands or nectar. Pollen pellets are colorful and range from light yellow to black [2]. Pollen grains have diverse morphological characteristics, such as cylindrical, round, triangular, bell-shaped or thorny characteristics [3]. The composition of bee pollen is closely related to the plant source and geographical location, as well as other related factors, such as soil type, beekeeper activities, and climatic conditions [1]. The main components of bee pollen are reducing sugars (40.7%, including 3.7% sucrose), protein (32.8%, including 11.5% essential amino acids), lipids (12.8%), bioelements (4.0%), vitamin C (0.19%), and β-carotene (0.07%) [2]. There is a wide assortment of commercial bee pollen products, so determining the quality of bee pollen in commercial products is required. However, only a few countries have officially standardized quality standards and limits of bee pollen and have permitted pollen use as a food additive; these countries include Argentina, Switzerland, Bulgaria, Poland, and Brazil [4, 5]. In ancient times, pollen has been applied as a food supplement or food diet additive and has been used for centuries as a kind of traditional medicine due to its nutritional and biological properties [6, 7]. The therapeutic and biological properties of bee pollen include antioxidant [8–12], anti-inflammatory [9], antimicrobial activities [10, 11] and antitumor [13, 14] activities; improvement of semen quality and fertility [15]; hypolipidemic and hepatoprotective activity [2]; and anti-atherogenic activity [16]. These potential activities of pollen vary with botanical and geographical origin [17, 18]. Assessment of the variety of beneficial biological properties and evaluation of the quality of bee pollen products has been a concern.

A previous study compared the sugar composition of bee pollen from China, South Korea, and Poland [19]. However, few studies have presented the biological activities of native South Korean bee pollen. In the current study, palynological identification of 18 bee pollen (BP) samples from *A. mellifera* collected from 16 different locations in South Korea was first analyzed. We also evaluated the biological features, including the antioxidant capacity based on 2,2-diphenyl-1-picrylhydrazyl (DPPH) free radical scavenging activity, inhibitory activities against human β-amyloid precursor cleavage enzyme (BACE1), human acetylcholinesterase (AChE), and antiproliferative activities toward five cancer cell lines (PC-3, MCF-7, A549, NCI-H727, and AGS), of bee pollen ethanol extracts (EBPs) for the first time. In addition, the growth inhibitory activities on four harmful intestinal bacteria, six lactic acid-producing bacteria, two nonpathogenic bacteria, and an acidulating bacterium were also assessed. In addition, the relationships between the total flavonoid and polyphenol contents and the biological properties of the 18 different EBP samples were also determined. The present study could provide insight into the influence of floral and geographical origin on the biological activities of bee pollen, which would contribute to the choice or establishment of a widely recognized quality standard for pollen products.

## Methods

### Reagents

Glycerol, quercetin, gallic acid, Folin-Ciocalteu’s phenol reagent, DPPH, ascorbic acid, 5,5-Dithiobis-(2-nitrobenzoic acid) (DTNB), acetylthiocholine iodide (ATChI), 3-(4,5-dimethylthiazol-2-yl)-2,5-diphenyl tetrazolium bromide (MTT), and anticancer agent cisplatin were purchased from Sigma-Aldrich (St. Louis, MO). Recombinant human BACE1, human AChE, and fluorogenic peptide substrate (FPS) McaSEVNLDAEFRK (Dnp) RR-NH_2_ were purchased from R&D system (Minneapolis, MN). Brain Heart Infusion (BHI) broth and Eggerth-Gagnon agar were purchased from Becton, Dickinson and Company (Sparks, MD) and Eiken Chemical (Tokyo, Japan), respectively. Minimum essential medium (MEM), RPMI 1640 medium, and fetal bovine serum (FBS) were purchased from Life Technologies (Grand Island, NY). All other reagents and chemicals used in the current study were available commercially, and with reagent-grade quality.

### Bee pollen samples and preparation of extract

Eighteen bee pollen samples (BP1-BP18) used for biological activities detection were purchased from bee keepers’ shops in 16 different locations from 6 provinces of South Korea. The localities and coordinates of these bee pollen samples are listed in Table 1. One bee pollen sample of China was supplied from bee keeper in Weihai City, Shandong Province. Total nineteen bee pollen samples were extracted three times with 70% ethanol at room temperature for 2 days each, and filtered. The combined extract solutions were concentrated or dryness by rotary evaporation at 40 °C under reduced pressure according to the previous study [20]. The yield of concentrated extracts was obtained from 46.85 to 63.83% (Table 1). And the ethanol extracts were kept at − 20 °C for further analysis.

**Table 1:** List of 18 bee pollen samples examined from 16 different locations of South Korea and the yield of their ethanol extract

Sample	Locality	Coordinate	Yield (%)
BP1	Yeoju (Gyeonggi)	37°22′16.2″N 127°35′45.2″E	56.07
BP2	Yeoju (Gyeonggi)	37°22′16.2″N 127°35′45.2″E	58.65
BP3	Yeoju (Gyeonggi)	37°22′16.2″N 127°35′45.2″E	56.48
BP4	Gapyeong (Gyeonggi)	37°49′53.55″N 127°30′35.58″E	60.44
BP5	Hwaseong (Gyeonggi)	37°11′58.18″N 126°49′52.28″E	49.20
BP6	Uiwang (Gyeonggi)	37°20′40.92″N 126°58′5.92″E	61.18
BP7	Paju (Gyeonggi)	37°45′35.53″N 126°46′48.64″E	63.83
BP8	Samcheok-si (Gangwon)	37°26′59.53″N 129°9′54.74″E	59.06
BP9	Yangyang-gun (Gangwon)	38°4′31.41″N 128°37′7.86″E	62.80
BP10	Goryeong-gun (Gyeongsangbuk)	35°43′34.11″N 128°15′46.63″E	58.93
BP11	Andong (Gyeongsangbuk)	36°34′6.08″N 128°43′45.69″E	46.85
BP12	Gunwi-gun (Gyeongsangbuk)	36°14′34.25″N 128°34′21.99″E	54.73
BP13	Jeongeup (Jeollabuk)	35°34′11.59″N 126°51′21.22″E	55.74
BP14	Sancheong-gun (Gyeongsangnam)	35°24′56.12″N 128°52′24.59″E	51.68
BP15	Yeosu (Jeollanam)	34°45′37.35″N 127°39′44.00″E	54.33
BP16	Jindo (Jeollanam)	34°29′12.74″N 126°15′48.55″E	55.40
BP17	Damyang-gun (Jeollanam)	35°19′16.10″N 126°59′17.40″E	61.32
BP18	Hampyeong (Jeollanam)	35°03′57.38″N 126°30′59.59″E	58.77
CNBP	Weihai (Shandong)	36°41′38.66″N 121°11′3.84″E	54.96

*CNBP* Chinese bee pollen

### Palynological analysis of bee pollen

Pollen analysis was based on the method reported previously [21]. 2 g of commercial bee pollen samples were added into 15 mL Falcon tube, followed with mixed with 70% ethanol to complete 13 mL, and left for 30 min. Then the samples were ultrasonication for 5 min, and centrifuged for 3 min at 1500 rpm, and the sediment obtained was extracted with 70% ethanol and ultrasonicated again. A solution of glycerol and distilled water (v/v = 1:1) was added to the sediment to make the volume 13 mL, and suspended, then left for about 30 min. One drop of well-mixed bee pollen suspension was transferred on a microscope slide, covered with a cover slide and named, sealed with nail polish, and were analyzed under a microscope with a 400 × magnification.

### Determination of total flavonoid and phenolic contents

Total flavonoid contents in EBP samples were determined by AlCl_3_ colorimetric method based on our previous study [22]. The absorbance was determined at 435 nm using a VersaMax microplate reader with SoftMax Pro 5 Software. EBPs were evaluated at the final concentration of 1000 μg mL^**− 1**^. Quercetin (0–12.5 μg mL^**− 1**^) was applied as the standard, and the standard curve was Y = 0.1774 X + 0.0433 (correlation coefficient R^2^ = 0.9995), where X is the quercetin concentration in μg mL^**− 1**^, and Y is the absorbance measured at 435 nm. Total flavonoid contents were calculated by linear regression analysis based on the standard curve of quercetin and expressed as mg of quercetin equivalents (QE) per g of EBP samples concentrated and dry by rotary evaporation at 40 °C under reduced pressure.

Total phenolic contents in EBP samples were determined based on Folin–Ciocalteu colorimetric method described previously [22]. The absorbance was determined at 735 nm. EBPs were evaluated at the final concentration of 500 μg mL^**− 1**^. Gallic acid (0–62.5 μg mL^**− 1**^) was used as the standard, and the standard curve was Y = 0.2265 X + 0.0888 (R^2^ = 0.9950), where X is the gallic acid concentration in μg mL^**− 1**^ and Y is the absorbance of reaction mixture at 735 nm. Total polyphenol contents were calculated according to linear regression analysis based on the standard curve, and expressed as mg of gallic acid equivalents (GAE) per g of EBP samples prepared by the above method.

### DPPH radical scavenging assay

Antioxidants with natural origin, particularly in food applications, are considered to be widely used to prevent disease and healthy maintenance [23]. Bee pollen, or as a dietary supplement, is widely intake in daily life. Antioxidant activity of EBP was assessed by DPPH radical scavenging assay according to the method described in our previous study [22]. In order to obtain the EC_50_ value of radical scavenging activity of DPPH for each EBP sample, seven concentrations were applied with ranging from 15.6 to 2000 μg mL^**− 1**^. Ascorbic acid was used as a positive control and similarly formulated. The radical scavenging ability of each EBP sample was calculated according to the equation: % DPPH free radical scavenging activity = (1 – A_s_/A_c_) × 100, where A_c_ is the absorbance of the control and A_s_ is the absorbance of the sample.

### Fluorescence resonance energy transfer enzyme assay

In the amyloid precursor protein (APP) amyloidgenic pathway, BACE1 plays an important role in the generation of β-amyloid (Aβ) that the main composition of amyloid plaque related to the pathogenesis of AD [24]. BACE1 inhibitor has been regarded as a therapeutic target in reducing Aβ production. The BACE1 inhibitory activity of all EBP samples was evaluated by the fluorescence resonance energy transfer (FRET) enzyme assay method according to our previous studies [22, 25]. In order to get the EC_50_ of BACE1 inhibitory activity, seven concentrations were applied with ranging from 15.6 to 2000 μg mL^**− 1**^. The inhibition percentage of EBP sample against human BACE1 was calculated based on the formula: % inhibition = 100 – [(F_S_ – F_S0_)/(F_C_ – F_C0_)] × 100, where F_S_ and F_S0_ are the fluorescence indensity of samples at 60 min and zero time, and F_C_ and F_C0_ are that of control at 60 min and zero time, respectively.

### Acetylcholinesterase (AChE) inhibition assay

Acetylcholine (ACh), as a neurotransmitter plays an important role in learning and memory processes [26]. The AChE exists in the synaptic cleft of neurons and hydrolyzes ACh to reduce the level of it. In the brain of AD, the more decreased ACh level induced the memory deficits. AChE inhibitor increased the concentration of ACh in the neuron synapses by binding to the enzyme [27]. The AChE inhibition assay procedure was performed using the recombinant human AChE according to the manufacture’s protocol as described in our previous study [22]. The inhibition percentage of EBP sample against human AChE was calculated based on the equation: % inhibition = 100 – [(A_S_ – A_S0_)/ (A_C_ – A_C0_)] × 100, where A_S_ and A_S0_ are the absorbance of samples at 60 min and zero time, and A_C_ and A_C0_ are that of control at 60 min and 0 time, respectively.

### Human intestinal bacterial strains and growth inhibitory assay

It is well known that the intestinal microorganisms in human gastrointestinal tract plays important role in modulating various physiologic and metabolic function for human health [28, 29]. Our previous study has reported that bee product (bee propolis) collected from South Korea have beneficial effects on human intestinal bacteria [22]. There are a few literatures that deal with the relationship between bee pollen and human intestinal bacteria. In our study, to assess the effect of EBP samples on human intestinal bacteria, beneficial six lactic acid-producing bacteria employed in the food industry including *Bifidobacterium bifidum* ATCC 29521, *Bifidobacterium breve* ATCC 15700, *Bifidobacterium infantis* ATCC 25962, *Bifidobacterium longum* ATCC 15707, *Lactobacillus acidophilus* ATCC 4356, and *Lactobacillus casei* ATCC 393, and an acidulating bacterium (*Clostridium butyricum* ATCC 25779), as well as four harmful bacteria including *Clostridium difficile* ATCC 9689, *Clostridium paraputrificum* ATCC 25780, *Clostridium perfringens* ATCC 13124, *Staphylococcus aureus* ATCC 12600, and two nonpathogenic bacteria *(Bacteroides fragilis* ATCC 25285, and *Escherichia coli* ATCC 11775) were tested in the current study. All of these strains were purchased form American type culture collection (ATCC). Stock cultures of the bacterial strains were prepared with BHI broth (pH 7.6) containing 25% glycerol (v/v) and stored at − 70 °C. The cultures of *Staphylococcus aureus* ATCC 12600 and *Escherichia coli* ATCC 11775 were incubated at 37 °C for 24 h under aerobic condition, while the cultures of the other bacterial strains were incubated at 37 °C for 24 h in an atmosphere of 5% H_2_, 15% CO_2_, and 80% N_2_ in a FA-6 anaerorator (serial no. 98072851, Hirayama, Tokyo, Japan) according to the previous study [22]. The minimal inhibitory concentrations (MIC) of all EBP samples toward the organisms were determined by a microtiter plate-based bioassay in sterile 96 well plates, as described previously [30].

### Cancer cell lines and cell proliferation assay

Bee pollen as a natural and healthy food, can improve human immunity. Previous study has demonstrated pollen could markedly suppress tumor growth [13]. However, this is few study illuminated the anti-cancer effects of bee pollen collected from South Korea. There are five human cancer cell lines used in the present study including PC-3 (human prostate adenocarcinoma cell line), MCF-7 (human breast adenocarcinoma cell line), AGS (human stomach cancer), and NCI-H727 (human lung carcinoma cell line) supplied by the Korean Cell Line Bank (Seoul, South Korea); A549 (human lung carcinoma cell line) purchased from the American Type Culture Collection (ATCC) (Manassas, VA). The PC-3, MCF-7, NCI-H727, and AGS cell lines were cultured with RPMI 1640 containing 10% FBS and 1% antibiotic, whereas A549 cell line was cultured with MEM containing 10% FBS, 1% antibiotic solution, and 1% glutamine. Cells were grown in SPL Life Science cell culture dishes under 5% CO_2_ and 95% air at 37 °C. The MTT assay was examined to evaluate the antiproliferative activity of all EBP samples toward the above five the human cancer cell lines as described previously [31]. To obtain the IC_50_ value of antiproliferative activity of each EBP sample toward five human cancer cell lines, six concentrations ranging from 0.78 to 25 mg mL^**− 1**^ were determined. The cell treated by different concentration of EBP samples were cultured for 48 h. The cultured plates were then washed with 100 μL PBS, followed by adding 100 μL medium containing 0.05% MTT and then incubated for 4–6 h at the same condition stated previously. Then MTT solution was removed and 200 μL DMSO was added to each well. Finally, the plate was shaken for 10 min at room temperature to dissolve the purple formazan crystals formed. The absorbance values were recorded at a 560 nm and a 670 nm reference by a VersaMax microplate reader stated above. Cisplatin served as positive controls and was similarly prepared. The inhibition of EBP sample to cells was calculated according to the following formula: % Inhibition = [1- (A_T_/A_C_)] × 100, where A_T_ is the absorbance value of EBP sample treated, and A_C_ for control.

### Statistical analysis

All the determination was carried out in at least triplicate. All the results were expressed as mean ± standard error (SE) of samples with three independent experiments. In each parameter, the differences between bee pollen samples were analyzed using one-way analysis of variance (ANOVA) followed by Bonferroni multiple-comparison method using SPSS 16.0 program (SPSS Inc. Chicago, IL, USA).

## Results

### Palynological identification of bee pollen samples

On the basis of palynological analysis, 4 of 18 pollen samples from South Korea (BP3, BP4, BP13 and BP15) were found to be monofloral and presented four dominant pollen types: *Quercus palustris*, *Actinidia arguta*, *Robinia pseudoacacia*, and *Amygdalus persica*. One sample (BP12) was found to be bifloral, relating to *Quercus palustris* and *Robinia pseudoacacia*. Most of the pollen samples, including BP1, BP2, BP5, BP6, BP7, BP8, BP9, BP10, BP11, BP14, BP16, BP17 and BP18, were found to be heterofloral. As a previous study reported, pollen samples with pollen type frequencies higher than 45% were considered the predominant pollen types, 16–45% frequency were considered secondary pollen types, and 3–15% were considered important minor pollen types [20]. In the current study, we found that *Quercus palustris* was dominant in the BP1, BP2, BP4, BP8, BP12, and BP15 samples, *Actinidia arguta* was dominant in the BP3 sample, *Amygdalus persica* was dominant in the BP11 sample, and *Robinia pseudoacacia* was dominant in the BP13 sample. However, no dominant pollen was observed in the BP5, BP6, BP7, BP9, BP10, BP14, BP16, BP17, and BP18 samples (Table 2).

**Table 2:** Comparison of 18 pollen load samples from *Apis mellifera* evaluated by a pool of 2 g of each bath. Only the pollen sample with frequency > 3% were considered

Sample	Time of collection	Pollen type identification	Evaluation of pollen sample
BP1	May	*Quercus palustris* (+++); *Robinia pseudoacacia* (++), *unknown* (+)	Heterofloral with domain of *Quercus palustris*
BP2	May	*Quercus palustris* (+++), *Robinia pseudoacacia* (++)	Heterofloral with domain of *Quercus palustris*
BP3	May	*Actinidia arguta* (++++)	Monofloral of *Actinidia arguta*
BP4	May	*Quercus palustris* (++++)	Monofloral of *Quercus palustris*
BP5		*Quercus palustris* (++), *Robinia pseudoacacia* (++), *unknown* (+), *Pinus* (+)	Heterofloral
BP6	June	*Robinia pseudoacacia* (++), *Quercus palustris* (++), *unknown* (+)	Heterofloral
BP7	June	*Robinia pseudoacacia* (++), *Quercus palustris* (++), *Pinus* (+)	Heterofloral
BP8	May	*Quercus palustris* (+++), *Robinia pseudoacacia* (++), *unknown* (+)	Heterofloral with domain of *Quercus palustris*
BP9	May	*Quercus palustris* (++), *Robinia pseudoacacia* (++), *Actinidia arguta* (++), *unknown* (+), *Pinus* (+)	Heterofloral
BP10	May	*Quercus palustris* (++), *Robinia pseudoacacia* (++), *Actinidia arguta* (++), *unknown* (+), *Pinus* (+)	Heterofloral
BP11	April	*Amygdalus persica* (+++), *Robinia pseudoacacia* (++) *Actinidia arguta* (++), *Taraxacum mongolicum* (+)	Heterofloral with domain of *Amygdalus persica*
BP12	April	*Quercus palustris* (+++), *Robinia pseudoacacia* (++)	Bifloral of *Quercus palustris* (dominant) and *Robinia pseudoacacia*
BP13	June	*Robinia pseudoacacia* (++++)	Monofloral of *Robinia pseudoacacia*
BP14	May	*Quercus palustris* (++), *Robinia pseudoacacia* (++), *Actinidia arguta* (+)	Heterofloral
BP15	May	*Quercus palustris* (++++)	Monofloral of *Quercus palustris*
BP16	May	*Quercus palustris* (++), *Robinia pseudoacacia* (++), *Actinidia arguta* (++), *Pinus* (+)	Heterofloral
BP17	May	*Robinia pseudoacacia* (++), *Actinidia arguta* (++), *Pinus* (+)	Heterofloral
BP18	April	*Amygdalus persica* (++), *Robinia pseudoacacia* (++), *Actinidia arguta* (++)	Heterofloral
CNBP	August	*Nelumbo nucifera* (++++)	Monofloral of *Nelumbo nucifera*

*CNBP* Chinese bee pollen

++++, circa > 85%: very frequent; +++, circa 45 to 85%: frequent; ++, circa 15 to 45%: few frequent; +, circa 3 to 15%: rare

### Total flavonoids and phenolic contents of bee pollen samples

**Fig. 1:**
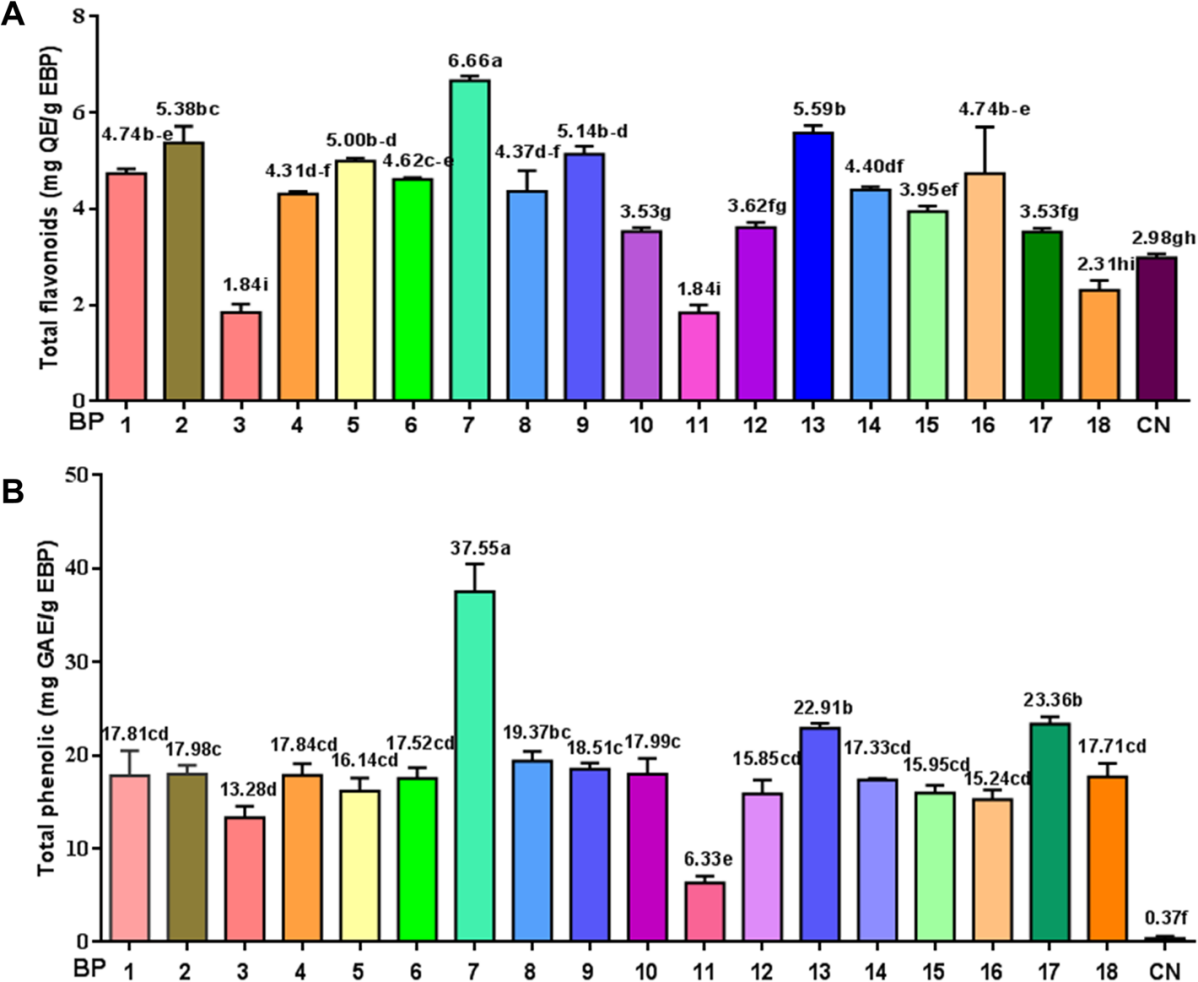
Total flavonoids (**a**) and phenolic content (**b**) in 18 different EBP samples from 16 different locations of South Korea, and 1 Chinese bee pollen ethanol extracts (mean ± SD). QE: Quercetin equivalent; GAE: gallic acid equivalent; EBP: ethanol extract of bee pollen. Means followed by the same letter in the column are not significantly different (*P* = 0.05, Bonferroni method)

The total flavonoid and phenolic compound contents of 18 EBP samples collected from 16 different regions of South Korea were compared with one Chinese EBP sample (Fig. 1). The total flavonoid contents ranged from 1.84 to 6.66 mg of quercetin equivalents per gram of EBP, while the flavonoid content of the Chinese bee pollen was 2.98 mg. Ethanol extract samples of BP7, BP13, BP2, BP9 and BP5 showed higher flavonoid contents (6.66–5.00 mg QE/g EBP) than those of other samples. The samples with the lowest total flavonoid contents were BP3 and BP11, with a value of 1.84 mg QE/g EBP. This result indicated that pollen predominantly from the plant species *Robinia pseudoacacia* contained a higher amount of flavonoids than that contained in pollens predominantly from *Actinidia arguta* and *Amygdalus persica*. The total phenolic compound contents of Korean EBP samples ranged from 6.33 to 37.55 mg of gallic acid equivalents per gram of EBP and were higher than the phenolic acid content of the Chinese sample (0.37 mg). Similar results were found in the phenolic compound content assay, in which the EBP7 and EBP13 samples presented higher phenolic compound contents than those of phenolic compounds in pollen from other regions, with values of 37.55 and 22.91 mg GAE/g EBP, respectively. The total flavonoids of the BP3 and BP11 samples, with values of 6.33 and 13.28 mg GAE/g EBP, respectively, were the lowest, which demonstrated that pollen grains predominantly from the plant species *Robinia pseudoacacia* possess higher amounts of flavonoids than those of flavonoids in pollen grains predominantly from *Actinidia arguta* and *Amygdalus persica*. The sample collected from China had a lower phenolic compound content than those of all the Korea BP samples, indicating that pollen from the plant species of *Nelumbo nucifera* contains low amounts of phenolic compounds. In addition, BP showed different amounts of phenolic and flavonoid contents varying from different geographical origins, which was consistent with a previous report that bee pollen collected by bees shows different contents of polyphenols due not only to its botanical origin but also to its geographical origin [17].

### DPPH free radical scavenging activity

Antioxidants of natural origin, particularly in food applications, are considered to be widely used to prevent disease and health maintenance [23]. The DPPH free radical scavenging assay system was used to test the antioxidant activity of EBP samples. The scavenging activity of the free DPPH radical was presented in terms of EC_50_, and the EC_50_ values of each EBP sample are listed in (Table 3). Based on the EC_50_ values, 16 Korean EBP samples showed antioxidant activity with EC_50_ values ranging from 292.0 to 673.9 μg mL^− 1^, which were lower than that of the positive ascorbic acid control (EC_50_, 13.8 μg mL^− 1^). Sample BP18 presented the highest antioxidant activity, with an EC_50_ value of 292.0 μg mL^− 1^, followed by sample BP7, with an EC_50_ value of 319.6 μg mL^− 1^. The ethanol extracts of samples BP3 and BP11 and the Chinese sample presented low antioxidant activity with EC_50_ values higher than 1 mg mL^− 1^. This indicates that BP predominantly from the plant species *Actinidia arguta* presented a lower DPPH radical scavenging ability than that BP predominantly from *Amygdalus persica*, *Robinia pseudoacacia*, and *Quercus palustris*. BP11 and BP18 were observed to be similar according to the palynological analysis, but they were found to have significant differences in terms of antioxidant effects. This indicated that a small number of pollen grains in the BP18 sample rather than in the BP11 may contribute to its DPPH radical scavenging ability. In addition to its botanical origin, other factors, including geographical origin and climate, affect BP antioxidant ability.

**Table 3:** Antioxidant and BACE1 inhibitory activity of 18 bee pollen samples examined from 16 different locations of South Korea and 1 sample from China

Sample No.	DPPH	BACE-1
	EC_50_, μg/mL	EC_50_, μg/mL
BP1	544.0 ± 53.22c	369.6 ± 0.58c
BP2	450.1 ± 10.45bc	642.1 ± 6.81 g
BP3	> 1000	669.5 ± 3.02 g
BP4	673.9 ± 20.99d	1008.9 ± 8.09 k
BP5	525.7 ± 63.87c	497.5 ± 5.90e
BP6	408.0 ± 24.92abc	414.7 ± 10.30d
BP7	319.6 ± 15.48ab	236.0 ± 3.01a
BP8	452.7 ± 28.49bc	635.6 ± 8.65 g
BP9	403.7 ± 11.91abc	881.1 ± 5.28j
BP10	534.0 ± 34.03c	1491.7 ± 17.95 m
BP11	> 1000	760.5 ± 6.99 h
BP12	403.2 ± 21.83abc	1083.7 ± 13.37 l
BP13	447.3 ± 31.17bc	1004.1 ± 7.49 k
BP14	401.0 ± 30.27abc	511.0 ± 14.13e
BP15	487.5 ± 32.40c	851.2 ± 15.77i
BP16	483.9 ± 46.78c	584.3 ± 17.04f
BP17	408.4 ± 15.85abc	496.2 ± 10.26e
BP18	292.0 ± 13.05a	830.1 ± 4.47i
CNBP	> 1000	322.7 ± 8.00b

*CNBP* Chinese bee pollen

Different letter (a-l) in the same row indicate significant difference *(p < 0.05*)

### In vitro human BACE1 inhibitory activity

BACE1 is well known as one of the most important APP cleaving enzymes in AD pathology. BACE1 inhibitors have been regarded as therapeutic targets in reducing Aβ production, thereby alleviating AD. There is no report available demonstrating the relationship between pollen samples and BACE1 activity. In the current study, we first evaluated the potent BACE1 inhibitory activity of 18 Korean bee pollen samples in vitro according to the FRET enzyme assay method. As the results showed, 18 Korean bee pollen samples exhibited anti-BACE1 ability, with EC_50_ values ranging from 236.0 to 1492 μg mL^**− 1**^ (Table 3). Sample BP7 showed the highest activity (EC_50_, 236.0 μg mL^**− 1**^), followed by samples BP1 and BP6 with EC_50_ values of 369.6 and 414.7 μg mL^**− 1**^, respectively. BP13, BP4, BP12, and BP10 showed lower inhibitory activities than those of other samples, with EC_50_ values of 1004, 1009, 1084, and 1492 μg mL^**− 1**^, respectively, which were higher than 1000 μg mL^**− 1**^. These results demonstrated that the heterofloral pollen samples exhibited better anti-BACE1 activity than those of monofloral pollen. However, the Chinese monofloral bee pollen, with an EC_50_ of 322.7 μg mL^**− 1**^, indicated that *Nelumbo nucifera* pollen type was found to be a better candidate for anti-BACE1 than monofloral *Quercus palustris*, *Actinidia arguta*, *Robinia pseudoacacia*, and *Amygdalus persica* pollens. In addition, the bee pollen with *Robinia pseudoacacia* as the dominant source possessed a greater potential inhibitory ability against BACE1 than those of pollen with *Quercus palustris* as the predominant origin.

### Human AChE inhibitory activity

AChE inhibitors can increase the concentration of ACh in neuronal synapses in AD to promote damaged neurological recovery [27]. Some bee products, such as propolis, have been discovered to possess AChE inhibitory activities from different locations in Morocco and South Korea [22, 32]. However, few studies have reported the inhibitory activity of pollen against AChE. In the current study, the inhibitory activity of bee pollen against human AChE was assessed. However, the EC_50_ values were higher than 2 mg mL^− 1^, indicating that potential inhibitory activities were not strong in the 18 EBP samples from 16 different regions in South Korea.

### Human intestinal bacteria growth inhibitory activity

Bee pollen extracts have been previously reported to possess antimicrobial activity [10, 11]. The growth inhibitory activity of human intestinal bacteria was assessed in our study. The 18 EBP samples from Korea did not inhibit beneficial bacteria, including six lactic acid-producing bacteria (*Bifidobacterium bifidum* ATCC 29521, *Bifidobacterium breve* ATCC 15700, *Bifidobacterium infantis* ATCC 25962, *Bifidobacterium longum* ATCC 15707, *Lactobacillus acidophilus* ATCC 4356, and *Lactobacillus casei* ATCC 393) and an acidulating bacterium (*Clostridium butyricum* ATCC 25779), and did not show potent strong inhibitory activity toward two nonpathogenic bacteria *(Bacteroides fragilis* ATCC 25285 and *Escherichia coli* ATCC 11775). The MIC values of the 18 Korean and 1 Chinese bee pollen samples were > 50 mg mL^**− 1**^ toward four harmful bacteria (*Clostridium difficile* ATCC 9689, *Clostridium paraputrificum* ATCC 25780, *Clostridium perfringens* ATCC 13124, and *Staphylococcus aureus* ATCC 12600). There is limited literature on the antimicrobial activity of bee pollen in South Korea. The above results proved that the 18 bee pollen samples collected from South Korea in our study did not show considerable effects on beneficial, nonpathogenic, or harmful bacteria.

### Antiproliferative effect on cancer cell lines

There are few reports about the antiproliferative activity of bee pollen toward human cancer cell lines. Bee pollen polysaccharides from *Rosa rugosa* have been observed to possess antiproliferative effects on the human colorectal adenocarcinoma cell lines HT-29 and HCT116 [13]. In addition, bee pollen extracts from different solvents showed different antiproliferative activities against BT474, HepG2, KATO-III and SW620 cells [33]. In this study, the antiproliferative effect toward five human cancer cell lines of all 18 Korean EBP samples and 1 Chinese sample was measured and compared with that of the commercial anticancer agent cisplatin (Table 4). Based on the IC_50_ value, the antiproliferative effect on the PC-3 cell line ranged from 2.7 to 14.4 mg mL^**− 1**^. BP8 was the most active bee pollen sample, followed by BP5 and BP11, with IC_50_ values of 2.9 and 3.3 mg mL^**− 1**^, respectively. For the MCF-7 cell line, the IC_50_ value of 18 Korean bee pollen samples ranged from 0.9 to 12.7 mg mL^**− 1**^. Among them, the BP7 sample showed the highest activity, followed by BP2, BP1, and BP11, with IC_50_ values of 1.4, 1.5, and 1.9 mg mL^**− 1**^, respectively. The BP3 sample showed the lowest activity toward MCF-7 compared with the other samples, with an IC_50_ value of 12.7 mg mL^**− 1**^. Toward the A549 cell line, 16 Korean EBP samples presented antiproliferative effects, with IC_50_ values ranging from 5.0 to 20.6 mg mL^**− 1**^. BP7, BP5, BP6, and BP9 were the most active bee pollen samples, with IC_50_ values between 5.0 and 9.1 mg mL^**− 1**^. However, samples from BP3 and BP15 did not show significant antiproliferative effects. For the NCI-H727 cell line, the IC_50_ value of 18 the Korean samples was between 2.7 and 17.7 mg mL^**− 1**^. The BP2 sample was the most active (IC_50_ = 2.7 mg mL^**− 1**^), followed by BP5, BP9 and BP6, with IC_50_ values of 3.2, 3.4, and 3.9 mg mL^**− 1**^, respectively. Toward the AGS cell line, the IC_50_ value of the 18 Korean bee pollen samples ranged from 2.4 to 8.7 mg mL^**− 1**^; among them, BP11 was the most active, followed by BP7, with IC_50_ values of 2.4 and 2.5 mg mL^**− 1**^, respectively. Chinese samples showed anticancer effects toward PC-3, MCF-7, A549, NCI-H727, and AGS cancer cell lines with IC_50_ values of 3.3, 2.8, 16.1, 2.8, and 1.8 mg mL^**− 1**^, respectively. However, the antiproliferative effects of bee pollen samples toward five cancer cell lines were less potent than the anticancer agent cisplatin. The abovementioned results showed that EBP7 showed comparatively good activity toward five cancer lines, particularly PC-3, MCF-7, A549, and AGS. The EBP5 sample possessed better inhibitory activity toward four cancer lines, namely, PC-3, A549, NCI-H727 and AGS. However, BP3, a monofloral sample of *Actinidia arguta* pollen, was observed to show comparatively weak activity toward five cancer lines. BP4, BP12 and BP13 showed relatively weak inhibitory activity. Taken together, pollen grains with heterofloral botanical origin presented preferable inhibitory effects on cancer cell growth compared to those of pollen grains with bifloral and monofloral sources in the current study. However, the EBP sample from China, a monofloral pollen with a *Nelumbo nucifera* origin, presented good effects against PC-3, MCF-7, NCI-H727, and AGS cell lines but not against A549 cells. All the above data demonstrated that the anticancer effects of pollen samples vary with different botanical and geographical origins. In addition, the anticancer activity also depends on the cancer cell line. Therefore, EBPs might be used as a potential supplementary anticancer treatment agent, and specific pollen products can be suggest for different types of cancer.

**Table 4:** Antiproliferative activity of 18 different EBP samples from 16 regions of South Korea pollen ethanol extracts toward 5 cancer cell lines

Sample	PC-3 cell	MCF-7 cell	A549 cell	NCI-H727	AGS cell
	IC_50_, mg/mL	IC_50_, mg/mL	IC_50_, mg/mL	IC_50_, mg/mL	IC_50_, mg/mL
BP1	6.6 ± 0.32def	1.5 ± 0.17ab	15.1 ± 0.14cde	4.6 ± 0.54bcd	6.8 ± 0.05c
BP2	5.2 ± 0.21bcd	1.4 ± 0.13a	15.3 ± 0.05cde	2.7 ± 0.27a	6.9 ± 0.09c
BP3	7.0 ± 1.36ef	12.7 ± 0.55j	> 25	6.7 ± 0.79efg	6.7 ± 0.05c
BP4	14.4 ± 0.07i	5.1 ± 0.50fgh	18.5 ± 2.31ef	9.6 ± 0.24 h	6.8 ± 0.04c
BP5	2.9 ± 0.28a	3.7 ± 0.24cdef	8.0 ± 0.33b	3.2 ± 0.08ab	3.4 ± 0.09ab
BP6	9.9 ± 0.61 h	2.8 ± 0.13abcde	8.8 ± 0.46b	3.9 ± 0.05abc	3.4 ± 0.04ab
BP7	4.0 ± 0.09abc	0.9 ± 0.11a	5.0 ± 0.18a	4.8 ± 0.29bcd	2.5 ± 0.15ab
BP8	2.7 ± 0.29a	5.9 ± 0.55hi	13.2 ± 0.59 cd	5.4 ± 0.22cde	4.1 ± 0.20b
BP9	3.8 ± 0.31ab	3.4 ± 0.51bcdef	9.1 ± 0.68b	3.4 ± 0.24ab	3.4 ± 0.06ab
BP10	6.5 ± 0.45def	3.5 ± 0.54cdef	14.8 ± 0.38cde	9.2 ± 0.37 h	6.1 ± 0.59c
BP11	3.3 ± 0.12ab	1.9 ± 0.20abc	12.6 ± 0.38c	5.2 ± 0.46cde	2.4 ± 0.16ab
BP12	3.4 ± 0.19ab	4.5 ± 0.64efgh	14.9 ± 1.56cde	17.7 ± 0.33 k	8.7 ± 1.64d
BP13	8.9 ± 0.09gh	6.8 ± 0.46i	17.0 ± 0.63def	10.9 ± 0.17i	8.0 ± 0.27 cd
BP14	6.9 ± 0.08def	3.9 ± 0.40defg	17.8 ± 0.14ef	7.1 ± 0.21 fg	6.1 ± 0.01c
BP15	8.1 ± 0.33 fg	5.5 ± 0.93ghi	> 25	12.8 ± 0.79j	6.7 ± 0.13c
BP16	5.7 ± 0.6719cde	2.4 ± 0.20abcd	20.6 ± 1.13f	11.5 ± 0.40i	7.2 ± 0.07c
BP17	4.2 ± 0.18abc	2.3 ± 0.21abcd	12.0 ± 0.35c	6.0 ± 0.57dfe	6.0 ± 0.20c
BP18	4.3 ± 0.14abc	2.3 ± 0.06abcd	19.9 ± 1.91f	7.8 ± 0.38 g	3.7 ± 0.07b
CNBP	3.3 ± 0.18ab	2.8 ± 0.31abcde	16.1 ± 0.11cde	2.8 ± 0.11a	1.8 ± 0.04a
Cisplatin	0.081 ± 0.0053	0.005 ± 0.0022	0.022 ± 0.0006	0.004 ± 0.0003	0.035 ± 0.0015

*CNBP* Chinese bee pollen

Different letter (a-k) in the same row indicate significant difference *(p < 0.05*)

## Discussion

Bee pollen consists of phenolic and flavonoid compounds that are related to serious therapeutic activities, including antioxidant, antibiotic, antidiarrhoeic and antineoplasic properties [34]. The total phenolic and flavonoid contents in BP have been determined in many previous studies. The total phenolic (TP) and total flavonoid content (TF) was reported in bee pollen from Brazil (TP, 41.5–213.2 mg g^− 1^) [20], from the southern region of Brazil (TP, 19.28–48.90 mg g^− 1^; TF, 2.10–28.33 mg g^− 1^) [35], from Northwest Algeria (TP, 30.46 mg g^− 1^; TF, 8.92 mg g^− 1^) [36], from the Baltic region (TP, 24.1–45.5 mg g^− 1^; TF, 6.1–11.6 mg g^− 1^) [37], from Portugal (TP, 10.5–16.8 mg g^− 1^) [38], from Venezuela (TP, 3.96–12.867 mg g^− 1^) [39], and from Greece (TP, 10.49 mg g^− 1^) [10]. In the present study, the flavonoid and phenolic compound contents of 18 EBP samples from South Korea ranged from 1.84 to 6.66 mg g^− 1^ and from 6.33 to 37.55 mg g^− 1^, respectively. However, the total phenolic and flavonoid compound contents of bee pollen stated in previous studies varied from 3.96 to 213.2 mg g^− 1^ and from 2.1 to 28.33 mg g^− 1^, respectively. Most of the EBP samples contained a higher amount of flavonoid than the amounts in the Chinese bee pollen sample analyzed in this study, and all of the Korean bee pollen samples showed phenolic compound contents that were 17- to 101-fold higher than those of the Chinese sample assessed in the current study. The phenolic compound and flavonoid composition in bee pollen has been well documented by Bridi et al. [18], who reported that three phenolic acids (syringic acid, coumaric acid, and cinnamic acid) and one flavonoid (myricetin) were detected in all 23 pollen samples. In addition, ferulic acid, cinnamic acid, quercetin, apigenin, and rhamnetin were also observed in most samples in considerable amounts. The chemical characterization of the phenolic compounds and flavonoids in our samples need to be further studied.

According to our results, we first demonstrated that pollen grains collect in South Korea that were predominantly from the plant species *Robinia pseudoacacia* possessed higher phenolic compound and flavonoid contents than those of pollen grains predominantly from *Actinidia arguta* and *Amygdalus persica*. However, pollen from the plant species *Nelumbo nucifera* rarely contained phenolic compounds. Therefore, phenolic compounds and flavonoids in bee pollen vary with botanical and geographical origins, which was consistent with a previous study [17].

DPPH radicals have been widely used to determine the free radical scavenging capacity of various samples. EC_50_, which is the amount of an antioxidant needed to inhibit 50% of the initial DPPH concentration, was used to measure the radical scavenging ability. Relatively low EC_50_ values indicated high radical scavenging activity of the EBP samples. For the DPPH radical scavenging capacity, the EC_50_ values have been reported to range from 40 to over 500 μg mL^**− 1**^ in pollen samples collected from New Zealand and Portugal [40], from 810 to 4690 μg mL^**− 1**^ in pollen samples from the southern region of Brazil [35], and from 2.16 to 5.87 mg mL^**− 1**^ in pollen samples from Portugal [38]. In the current study, the EC_50_ values of the 18 different EBP samples from Korea varied from 292.0 to over 1000 μg mL^**− 1**^, and the EC_50_ values of bee pollen stated above are between 40 and 5870 μg mL^**− 1**^. Aside from samples BP3 and BP11, most of the Korean bee pollen presented better DPPH radical scavenging capacity than that of the Chinese sample assessed in this study. The current study indicated that Korean bee pollen samples from *Amygdalus persica*, *Robinia pseudoacacia*, and *Quercus palustris* presented better antioxidant activity than that of pollen from *Actinidia arguta* and *Nelumbo nucifera*. Therefore, based on our study, the appropriate origin of collected bee pollen should be chosen to maximize its antioxidant function.

Amyloid plaque is the accumulation of Aβ, which is one of the major neuropathological hallmarks of Alzheimer’s disease (AD). BACE1 is the major β-secretase acting in the brain during the abnormal APP amyloidogenic processing pathway. The cholinergic deficit in the cerebral cortex and basal forebrain results in the cognitive impairment of patients with AD, and AChE hydrolyzes acetylcholine into choline and acetate, which then reduces acetylcholine [41]. BACE1 inhibitors that reduce the formation of Aβ and AChE inhibitors that increase cholinergic transmission have become two major targets in AD treatment [41, 42]. However, there is no information available concerning bee pollen extract inhibitory activity against human BACE1. In the current study, it was first reported that bee pollen extract possesses human BACE1 inhibitory activity, with EC_50_ values ranging from 236.0 to 1492 μg mL^**− 1**^. Based on these data, the heterofloral bee pollen sample exhibited better anti-BACE1 activity than that of the monofloral pollen samples. In addition, bee pollen with *Robinia pseudoacacia* as the dominant source possessed a better potential inhibitory capacity against BACE1 than that of bee pollen with *Quercus palustris* as the predominant origin. Therefore, *Robinia pseudoacacia*-derived pollen could be used as the most potential candidate for anti-BACE1 activity for AD prevention or treatment. A previous study reported that bee pollen of different geographical origins in Bahia, Brazil, presented AChE inhibitory activity with EC_50_ values of 3.93–967.53 μg mL^**− 1**^ [43], but unfortunately, in our study, all bee pollen samples did not present potent inhibitory activity against human AChE at a concentration of 2 mg mL^**− 1**^. A previous study reported that flavonoids and phenolic compounds derived from natural products showed potent anti-AChE capacity [44]. Bee pollen extracts contain abundant flavonoids and phenolic compounds. Anti-AChE activities were not observed in our samples; perhaps the constituents in these samples had antagonistic effects against AChE. Further constituent isolation and identification of those with inhibitory activity on AChE should be further studied. Taken together, the results suggest bee pollen extracts could have merit as potential anti-AD agents.

Some previous studies have indicated the antiproliferative effects of bee pollen extracts on cancer. A previous study reported that the IC_50_ value of bee pollen from Malaysia on MCF-7 cells was 15 mg mL^**− 1**^ [33]. The cytotoxic activities of Indonesian bee pollen extracts towards the five different cancer cell lines BT474, ChaGo, HepG2, KATO-III, and SW620 varied not only with the solvent used in the extraction but also the bee species and bee product source. Most of the bee pollen extracts did not show cytotoxic activity at a concentration of 20 μg mL^**− 1**^ [33]. Bee pollen polysaccharides from *Rosa rugosa* (WRPP) collected from China have been reported to possess dose-dependent antiproliferative activities with concentrations from 0 to 5 mg mL^**− 1**^, and WRPP showed approximately 70% cytotoxic activity toward HT-29 and HCT116 cancer cell lines at a concentration of 5 mg mL^**-1** 13^. The antiproliferative effect of German bee pollen samples showed high efficiency, reaching approximately 60 to 70% inhibition at a concentration of 1 mg mL^**− 1**^ against C26 mouse colon tumor cells [45]. In the present study, the antiproliferative effects of the 18 Korean EBP samples varied according to the source, which was consistent with a previous study [36]. All 18 Korean EBP samples showed potent cytotoxic effects on the human cancer cell lines PC-3, MCF-7, A549, NCI-H727, and AGS, with IC_50_ values ranging from 2.7 to 14.4 mg mL^**− 1**^, 0.9 to 12.7 mg mL^**− 1**^, 5.0 to > 25 mg mL^**− 1**^, 2.7 to 17.7 mg mL^**− 1**^, and 2.4 to 8.7 mg mL^**− 1**^, respectively. The anticancer effects of the bee pollen samples varies with botanical and geographical origins. In addition, the anticancer activity also depended on the cancer cell species. Therefore, EBPs might be useful as potential supplementary anticancer treatments and should be chosen in accordance with the cancer to be treated.

The correlation between biological features and phenolic compound or flavonoid contents has been well studied. Some studies have demonstrated a higher correlation between antioxidant and flavonoid and phenolic compound contents. Mesquite pollen extracts showed antioxidant capacity that was related to the flavonol concentration. Radical scavenging activity correlated with the total content of phenolic compounds, while the correlation coefficient was 0.95 [32, 37]. However, in the current study, correlation coefficient (*R*) analysis showed that no significant relation between the phenolic compound or flavonoid contents and antioxidant activity (*R*^*2*^ = 0.158 or 0.00005897, respectively) was observed, which was consistent with previous studies [38, 46]. In addition, it has been reported that the antioxidant activity varies with the solvent used to extract pollen samples, and there is no clear association with their phenolic compound contents [47]. The correlation between phenolic compound content and antioxidant activity is still controversial for bee pollen.

**Fig. 2:**
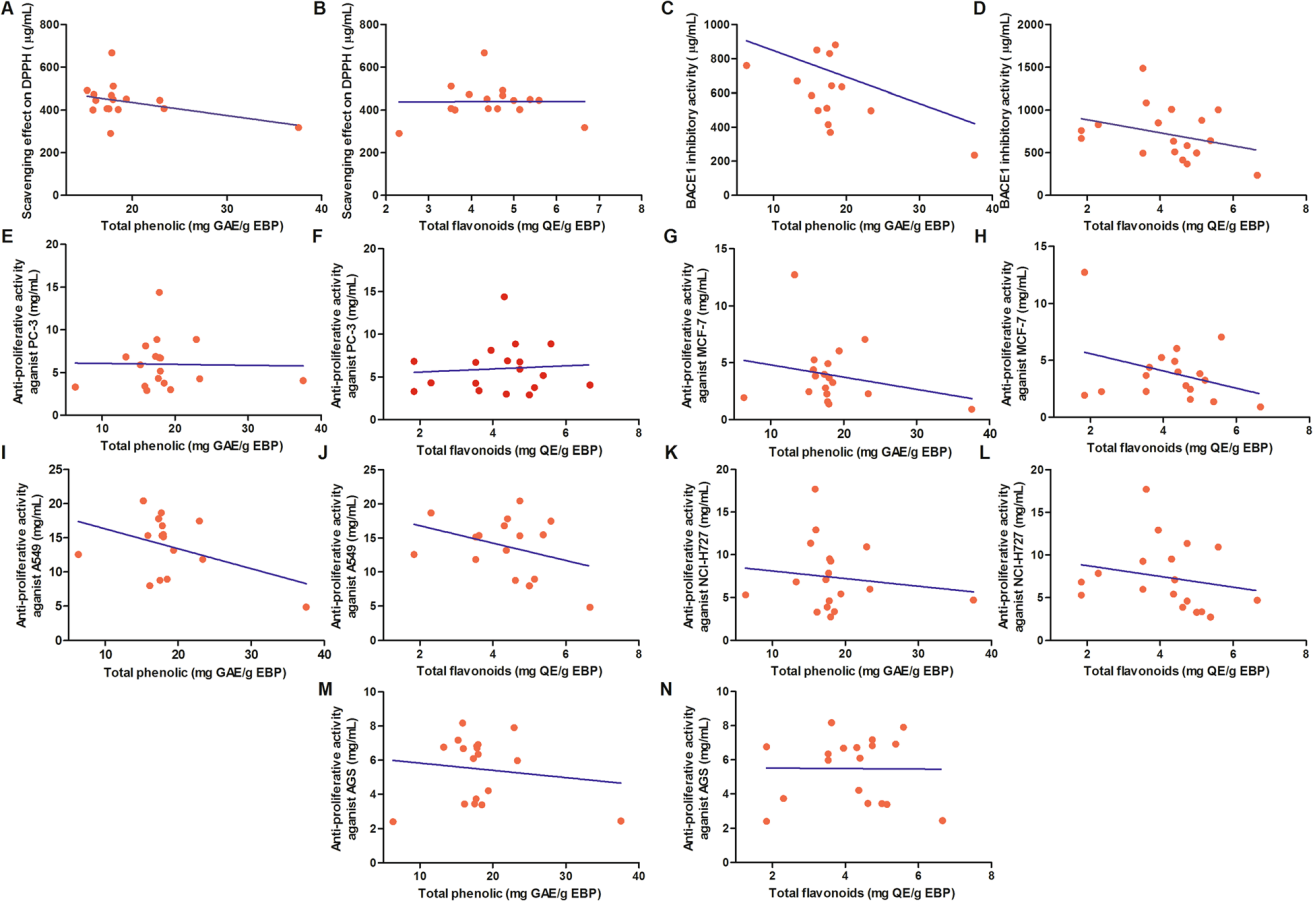
Pearson correlation coefficients for total phenol or flavonoid content and IC_50_ values of EBP samples biological activities. **a** Pearson correlation coefficients for total phenolic content and DPPH free radical scavenging activity (R^2^ = 0.158). **b** Pearson correlation coefficients for total flavonoids content and DPPH free radical scavenging activity (R^2^ = 0.00005897). **c** Pearson correlation coefficients for total phenolic content and BACE-1 inhibitory activity (R^2^ = 0.09420). **d** Pearson correlation coefficients for total flavonoids content and BACE-1 inhibitory activity (R^2^ = 0.1034). **e** Pearson correlation coefficients for total phenolic content and antiproliferative activity against cancer cell line PC-3 (R^2^ = 0.006436). **f** Pearson correlation coefficients for total flavonoids content and antiproliferative activity against cancer cell line PC-3 (R^2^ = 0.0005091). **g** Pearson correlation coefficients for total phenolic content and antiproliferative activity against cancer cell line MCF-7 (R^2^ = 0.05512). **h** Pearson correlation coefficients for total flavonoids content and antiproliferative activity against cancer cell line MCF-7 (R^2^ = 0.1239). **i** Pearson correlation coefficients for total phenolic content and antiproliferative activity against cancer cell line A549 (R^2^ = 0.1748). **j** Pearson correlation coefficients for total flavonoids content and antiproliferative activity against cancer cell line A549 (R^2^ = 0.1243). **k** Pearson correlation coefficients for total phenolic content and antiproliferative activity against cancer cell line NCI-H727 (R^2^ = 0.01843). **l** Pearson correlation coefficients for total flavonoids content and antiproliferative activity against cancer cell line NCI-H727 (R^2^ = 0.04156). **m** Pearson correlation coefficients for total phenolic content and antiproliferative activity against cancer cell line AGS (R^2^ = 0.01843). **n** Pearson correlation coefficients for total flavonoids content and antiproliferative activity against cancer cell line AGS (R^2^ = 0.00009681)

In addition, there was no observed direct correlation between BACE1 inhibitory activity (R^2^ = 0.09420, or 0.1034, respectively) or antiproliferative capacity against PC-3 (R^2^ = 0.006436, or 0.0005091, respectively), MCF-7 (R^2^ = 0.05512 or 0.1239, respectively), A549 (R^2^ = 0.1748, or 0.1243, respectively), NCI-H727 (R^2^ = 0.01843, or 0.04156, respectively), and AGS (R^2^ = 0.01843, or 0.00009681, respectively) and total phenolic compound or flavonoid contents in the EBP samples (Fig. 2). This indicated that there are some other bioactive constituents, such as phytosterols, phospholipids, fatty acids, and organic carotenoid pigments, that may be responsible for the biological properties of bee pollen [48]. The main constituents imparting these biological properties and health-promoting effects should be further isolated and identified.

## Conclusion

The ethanol extracts of bee pollen collected from different locations in South Korea has different DPPH radical scavenging activities and BACE1 in vitro inhibitory activities. In addition, Korean bee pollen ethanol extracts exhibited inhibitory effects on five human cancer cell lines. Fundamentally, Korean bee pollen-derived preparations could be considered nutritional addition to food that could prevent various diseases related to free radicals, neurodegenerative problems and cancer. This study demonstrated the potential anti-BACE1 activity of EBPs based on an in vitro assay system for the first time. However, in the current study, there was no significant relation between the phenolic compound or flavonoid contents and the antioxidant activity and anti-BACE1, and anticancer effects. Because the biological activities of bee pollen depend on the plant source and environmental conditions, such as geographical location, identification and selection, high-quality bee pollen is needed. In conclusion, our findings provide a scientific basis for evaluating the biological activities of bee pollen samples collected from several parts of South Korea and varied with botanical source. These findings might be used as a reference for consumer choices, bee pollen production, and industrial exploration to maintain human health.

## Data Availability

The datasets used and/or analyzed in the current study are available from the corresponding author with a reasonable request.

## Abbreviations

BP: Bee pollen
EBP: Ethanol extract of pollen
Anti-BACE1: β-amyloid precursor cleavage enzyme
AChE: Acetylcholinesterase
DPPH: 2,2-diphenyl-1-picrylhydrazyl
DTNB: 5,5-Dithiobis-(2-nitrobenzoic acid)
ATChI: Acetylthiocholine iodide
MTT: 3-(4,5-dimethylthiazol-2-yl)-2,5-diphenyl tetrazolium bromide
FBS: Fluorogenic peptide substrate
ATCC: American type culture collection
BHI: Brain Heart Infusion
MEM: Minimum essential medium
FBS: Fetal bovine serum
QE: Quercetin equivalents
GAE: Gallic acid equivalents
TP: Total phenolic
TF: Total flavonoids content
AD: Alzheimer’s disease
APP: Amyloid precursor protein (APP)
Aβ: β-amyloid
WRPP: Pollen polysaccharides from Rosa rugose
